# Dental-Pulp Stem Cells as a Therapeutic Strategy for Ischemic Stroke

**DOI:** 10.3390/biomedicines10040737

**Published:** 2022-03-22

**Authors:** Chikako Nito, Satoshi Suda, Yuko Nitahara-Kasahara, Takashi Okada, Kazumi Kimura

**Affiliations:** 1 Department of Neurological Science, Graduate School of Medicine, Nippon Medical School, Tokyo 113-8603, Japan; suda-sa@nms.ac.jp (S.S.); k-kimura@nms.ac.jp (K.K.); 2 Collaborative Research Center, Laboratory for Clinical Research, Nippon Medical School, Tokyo 113-8603, Japan; 3 Division of Molecular and Medical Genetics, The Institute of Medical Science, The University of Tokyo, Tokyo 108-8639, Japan; y-kasahara@g.ecc.u-tokyo.ac.jp (Y.N.-K.); t-okada@ims.u-tokyo.ac.jp (T.O.)

**Keywords:** dental pulp stem cells, cell-based therapy, neuroprotection, cerebral ischemia, clinical trials

## Abstract

Regenerative medicine aims to restore human functions by regenerating organs and tissues using stem cells or living tissues for the treatment of organ and tissue defects or dysfunction. Clinical trials investigating the treatment of cerebral infarction using mesenchymal stem cells, a type of somatic stem cell therapy, are underway. The development and production of regenerative medicines using somatic stem cells is expected to contribute to the treatment of cerebral infarction, a central nervous system disease for which there is no effective treatment. Numerous experimental studies have shown that cellular therapy, including the use of human dental pulp stem cells, is an attractive strategy for patients with ischemic brain injury. This review describes the basic research, therapeutic mechanism, clinical trials, and future prospects for dental pulp stem cell therapy, which is being investigated in Japan in first-in-human clinical trials for the treatment of patients with acute cerebral ischemia.

## 1. Introduction

Stroke is the second leading cause of death worldwide and constitutes a major socioeconomic problem [1]. Ischemic stroke accounts for 87% of all stroke cases, and is the leading cause of severe disability [2]. In the acute phase of brain ischemia, the neurological symptoms can be markedly improved by reopening the occluded blood vessel early after the onset of ischemia; therefore, thrombolytic therapy and endovascular treatment using a catheter to mechanically collect the thrombus are used [3,4]. However, treatment during the acute stage of cerebral infarction is time-sensitive. For example, intravenous thrombolysis with tissue plasminogen activator has a therapeutic time window and must be administered within 4.5 h after the onset of cerebral infarction, and endovascular treatment must be performed within 24 h at the latest after onset. Even when a patient is transported to the hospital within this time period, thrombolysis and endovascular therapy is not always possible due to the time required for clinical investigations, taking patient’s history, aspects related to medical facilities, and procedural issues. Furthermore, patients undergoing recanalization therapies may present poor outcome, as the region that is potentially salvageable, termed the “stroke penumbra,” is very narrow. Therefore, there is a need to develop alternative therapeutic strategies for these patients.

Cell therapy is a novel treatment that aims to improve motor and cognitive disorders after stroke [5]. Induced pluripotent stem cells (iPS cells) and embryonic stem cells (ES cells) can differentiate into various cells and tissues (pluripotency) and have potential for use in the treatment for patients with cerebral infarction. However, there are ethical issues associated with the use of ES cells because they are derived from fertilized human eggs, and iPS cells carry a risk of tumor formation. Mesenchymal stem cells (MSCs), which are human somatic stem cells, have garnered attention due to their potential for practical use. MSCs have low immunogenicity, accumulate at the site of tissue injury, and secrete growth factors, cytokines, and bioactive substances to control inflammation. In addition to bone marrow [6], MSCs can be harvested from dental pulp [7], adipose [8], amnion [9], and umbilical cord blood [10]. Although MSCs express human leukocyte antigen (HLA) molecules, their expression levels are low and they do not express costimulatory molecules, such as CD80, CD86, and CD40, and thus do not stimulate nonself T cells [11]. This property is thought to allow MSCs with different HLAs to escape T-cell attack (rejection) even when they are administered to patients [12]. Currently, bone marrow-derived MSCs (BM-MSCs) are used for the treatment of the graft-versus-host disease (GVHD) after transplantation and ischemic stroke, and are also being investigated in clinical trials [13,14,15,16]. However, BM-MSCs are obtained by bone marrow puncture, which is an invasive procedure. Additionally, it can be difficult to obtain enough number of cells from elderly patients, and cell culture requires time. Dental pulp stem cells (DPSCs), obtained from human deciduous and wisdom teeth, have attracted attention as novel stem cells that can overcome these limitations [17]. A clinical study investigating DPSC transplantation for acute cerebral infarction is underway in Japan (ClinicalTrials.gov Identifier: NCT04608838).

Here, we review the basics of stem cell therapy, the therapeutic mechanisms, the ex vivo therapeutic approach, and the current status of ongoing clinical trials.

## 2. Dental Pulp Stem Cells

In 2000, Gronthos et al. discovered the pulp stem cells present in the pulp tissue [18]. Human dental pulp tissue is believed to be derived from neural crest cells that differentiate from neural ectoderm during the fetal life [19]. Because these cells originate from the nervous system, they may be useful for nerve regeneration therapy. Dental stem cells include deciduous, periodontal ligament, papillary, and pulp stem cells, with the exception of deciduous stem cells, which are derived from permanent teeth. Stem cells exfoliated from human deciduous teeth (SHED) are the youngest DPSCs and have the highest proliferative potential. DPSCs can also be obtained from orthodontically extracted teeth, such as primary teeth, first premolars, and third molars (wisdom teeth).

In addition to neurons, DPSCs can differentiate into bone, muscle, vascular endothelium, and adipose tissue, and may have potential use as a regenerative medicine for these tissues. DPSCs express cell-surface antigen markers, similar to BM-MSCs [18], and can differentiate into osteogenic cells, chondrocytes, and adipocytes [20]. However, DPSCs are less invasive than BM-MSCs when harvested [19], have a higher proliferative capacity (approximately 2–3 times higher) [21], and more potent in suppressing T-cell reactivity than BM-MSCs [19,22].

The efficacy of stem cell transplantation for cerebral ischemia using BM-MSCs or neural stem cells has been reported in preclinical studies [23,24,25,26]. In addition, neuroprotective and motor function-improving effects of DPSCs, which have similar properties to BM-MSCs, have also been reported using experimental cerebral ischemia models. However, further research is needed to establish the efficacy and safety of DPSC transplantation for the treatment of patients with stroke in clinical practice.

## 3. Therapeutic Effects of DPSCs in Cerebral Ischemia

### 3.1. Neuroprotective Effects of DPSCs in Experimental Cerebral Ischemia

In this section, we describe the results obtained from animal studies carried out prior to the start of the clinical trial. We have published a paper on brain protection using human DPSCs derived from first premolars extracted for orthodontic treatment in 2018, which is also discussed below [27]. Cultured human DPSCs are morphologically similar to human BM-MSCs and are similar in size (15–16 μm), and their cell surface antigen profiles are positive for CD29, CD73, CD90, and CD105 and negative for CD34 and CD45, confirming that these cells are MSCs [6,21,27]. Of the 12 studies listed in Table 1, the middle cerebral artery occlusion model was used in 10, all of which were reperfusion models, that is, transient focal cerebral ischemia models. In eight studies, DPSCs were administered intravenously after ischemia, and in two studies, intracerebral administration was chosen. The timing of cell administration after ischemia was immediate in two studies, 3 h in two studies, 4 h in one study, 24 h in six studies, and 5 days in one study, with half of the cells administered after 24 h. In all these studies, there was a significant reduction in the infarct volume in the DPSC implantation group and an improvement in motor and cognitive function compared with the control group. Besides three studies, the timing of DPSC administration was 24 h after cerebral ischemia in most studies, regardless of the route of administration (intravenous or intracerebral), suggesting a wide therapeutic window for these cells [28,29,30,31,32,33]. Using tissue immunostaining, we revealed that the expression of ionized calcium-binding adaptor molecule (Iba-1) in the cortical infarct border region and microglial activity were significantly suppressed in rats in the DPSC group [27,28].

In addition, interleukin (IL)-1β and tumor necrosis factor (TNF)-α levels in the ischemic hemisphere as well as in the serum were significantly decreased in rats in the DPSC group using ELISA, indicating a significant decrease in the levels of inflammatory cytokines in the brain and serum. Thus, in a rat model of transient focal cerebral ischemia, intravenous administration of DPSCs significantly reduced the infarct size and neuronal degeneration in the cortical infarct border region after reperfusion, and improved motor function, which may be mediated by suppression of inflammation in the brain [27,35]. Furthermore, in one study using the rat severe forebrain ischemia model, Kumasaka et al. showed the efficacy of intravenous DPSC administration at 3 h after reperfusion [34].

Few basic studies have investigated the effects of DPSC transplantation using models of cerebral ischemia. Previously, intracerebral or intraventricular administration was deemed as the optimal route for stem cell administration [28,29,39]. However, from 2017 to 2018, when our paper was published, a series of studies showed that intravascular administration of DPSCs in a rodent model of focal cerebral ischemia model reduced ischemic injury and improved motor function (Table 1) is a superior route for the treatment of stroke, especially in the acute phase because it is a rapid method of administration.

However, although intra-arterial administration can deliver a large number of cells to the target organ, there is an increased risk for microvascular embolization after cell transplantation. The incidence of microvascular occlusion or infarction following intra-carotid administration is correlated with the size of the administered cells [40,41]. Because DPSCs are similar in size to other MSCs, the risk for arterial microembolization can be reduced by transvenous, rather than intra-arterial, administration. Although the distribution of DPSCs in the body, when administered intravenously in a model of cerebral ischemia, has not been reported, they may have a similar distribution as BM-MSCs, which predominantly accumulate in the lung and spleen [42]. Hence, in intravenous administration, filteration of DPSCs into the pulmonary circulation after systemic administration must be considered.

### 3.2. Therapeutic Mechanism

Although the therapeutic mechanism of MSC transplantation for cerebral infarction has been investigated in preliminary research, few studies have examined the therapeutic effects of DPSCs, including those used for the treatment of acute cerebral ischemia. Pluripotent stem cells, such as DPSCs, have restricted pluripotency, and have demonstrated a favorable clinical safety profile with no increased risk observed for tumorigenesis [43,44]. Recent studies have shown that human BM-MSCs used for xenotransplantation can survive in injured central nervous system tissues and exert an immunomodulatory role by elevating anti-inflammatory cytokines and suppressing proinflammatory cytokines secreted by microglia and macrophages, without the use of immunosuppressive drugs [45,46,47]. In vivo studies have demonstrated that DPSCs function via a paracrine mechanism of action [48,49], which is regulated by the release of secreted factors, cytokines, chemokines, cell adhesion factors, and growth factors, such as stromal cell-derived factor-1 (SDF-1), nerve growth factor (NGF), brain-derived neurotrophic factor (BDNF), and vascular endothelial growth factor (VEGF) [50,51], The autocrine and paracrine effects of MSCs improve the host cell microenvironment and promote recovery after injury or disease. Notably, stem cell transplantation has been described as a cell-based cytokine therapy [52]. For example, IL-10 produced by inhibitory T cells reduces the neurotoxic effects of TNF-α and interferon (IFN)-γ in the ischemic brain [53], whereas systemic administration of DPSCs suppresses TNF-α and IL-1β, which induce inflammation in the serum and brain after ischemia-reperfusion [27]. In addition, VEGF, which is expressed on macrophages, neurons, glia, and vascular endothelial cells, promotes angiogenesis during tissue repair [54], and because DPSCs are secreted in high numbers, the angiogenic effect of DPSCs may also be related to their protective effect in the brain.

Post-transplantation human DPSCs differentiate into functional neural progenitor cells and neurons and migrate to the brain [55,56]; however, transplanted DPSCs have also been reported to migrate to the ischemic border region and differentiate into neurons and astrocyte-like cells [30]. These cells express neuronal and neural stem cell markers, including βIII-tubulin, doublecortin, nestin, and neurofilament [31].

However, in studies utilizing intracerebral administration, very few (2–3%) DPSCs were found to survive in the ischemic side of the brain, and migrated to the paracrine ischemic area; most differentiated into astrocytes rather than neurons. If surviving DPSC-derived cells differentiate and migrate, improvements in the motor function after transplantation may be controlled by the bystander effect rather than by the replacement or differentiation of these cells. Although the mechanisms underlying the neuroprotective effect of transplanted DPSCs remain unknown, these may be due, in part, to the anti-inflammatory and angiogenic effects of cytokines and neurotrophic factors.

**Figure 1:**
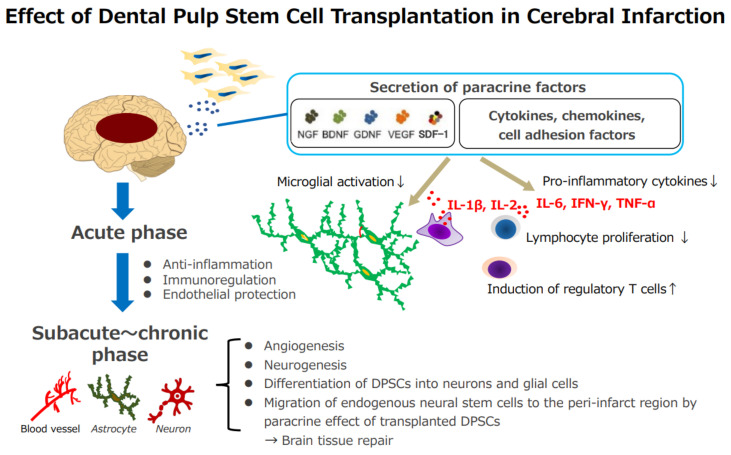
Effect of dental pulp stem cell transplantation in cerebral infarction. Acute phase: (1) direct secretion of stem cells or the accumulation of stem cells in the spleen; paracrine effects of various trophic factors and cytokines via the spleen, (2) the suppression of inflammatory cytokines, (3) protection of the vascular endothelium, (4) immune modulation through the induction of inhibitory T cells. Subacute~chronic phase: (1) promotion of angiogenesis, (2) promotion of intrinsic nerve regeneration, and differentiation of transplanted cells into neurons and glial cells. NGF, Nerve growth factor; BDNF, brain-derived neurotrophic factor; GDNF, glial cell-derived neurotrophic factor; VEGF, vascular endothelial growth factor; SDF-1, stromal cell-derived factor-1; IL-1β, interleukin 1 beta; IL-6, interleukin 6; IL-2, interleukin 2; IFN-γ, interferon gamma; TNF-α, tumor necrosis factor alpha.

In summary, the mechanisms underlying the therapeutic effects of DPSC transplantation in the acute phase of stroke are as follows (Figure 1): Direct secretion of paracrine factors by stem cells or the accumulation of stem cells in the spleen, paracrine effects of various trophic and fluid factors, such as cytokines, via the spleen, and the subsequent inhibition of inflammatory cytokines. Following the subacute phase, the following effects are possible: the promotion of angiogenesis, the promotion of intrinsic nerve regeneration, and the differentiation of transplanted cells into nerve and glial cells.

## 4. Enhancement of the Therapeutic Benefits of DPSCs by Genetic Modification

### 4.1. Genetic Modification of DPSCs

DPSCs may be a useful source of cells for the treatment of cerebral infarction, as shown in Table 1 [57,58,59]. However, there are some practical difficulties in the clinical use of DPSCs for cell therapy, including cell stability and survival, as well as functional limitations. To enhance the therapeutic effects of DPSCs, MSCs/DPSCs have been genetically modified using chemical and physical methods [60]. The delivery vectors into MSCs/DPSCs using chemical methods, such as cationic liposomes, are scalable and easy to synthesize and process; however, they present low transfection efficiency and induce transient expression. Gene transfection via electrical stimulation, such as electroporation and nucleofection, into MSCs/DPSCs has moderate transfer efficiency; however, it is limited by the loss of cells during the manipulation process. In contrast, DPSCs have been genetically modified using the CRISPR/Cas9-mediated gene editing system [61]. This has potential as a useful tool if it can overcome the problems caused by variants and off-target effects of gene editing. Genetically modified DPSCs have been effectively developed using viral vectors, including retroviral [62,63,64], lentiviral [65,66], adenoviral [67], and adeno-associated virus (AAV) vectors [35,68]. Lentiviral and retroviral vectors can be used to insert genes into MSCs/DPSCs with high transduction efficiency, although these vectors may induce off-target effects and activate oncogenes owing to insertional mutagenesis [60,69]. The adenovirus vector is effective when transient expression is required in MSCs/DPSCs [60,70]. Adenovirus vectors demonstrate low genotoxicity; however, there is a risk for cytotoxicity at high titers. AAV is markedly less immunogenic compared with adenoviral vectors [71,72]. Although AAV vectors have limited transport capacity, the stable gene expression system of these vectors is considered to be safe, with a low risk for genome insertion, random integration, off-target effects, and poor specificity [73,74,75,76]. AAV vector-mediated transgene expression is temporary, but can persist over a period of several months to several years [77] depending on the immune response and the turnover rate of infected cells [76,78].

### 4.2. Enhanced Therapeutic Benefits of Gene-Modified DPSCs on Post-Ischemia/Reperfusion Injury

Anti-inflammatory cytokines and growth factors have therapeutic potential for postischemia/reperfusion injury. We previously reported that transducing MSCs with an AAV vector expressing the anti-inflammatory cytokine, IL-10, enhanced their survival in a rat model of cerebral ischemia [42]. Improvements in survival, engraftment, and immune-modulation of MSCs were elucidated by stable expression of IL-10, suggesting that IL-10 promotes neuroprotection in an experimental model of acute ischemic stroke. Similar to MSCs, DPSCs transduced with the AAV/IL-10 vector could enhance cell survival and exert a paracrine effect after transplantation [68]. In fact, IL-10-expressing DPSCs demonstrated markedly reduced expression of proinflammatory monocyte chemotactic protein-1 (MCP-1) and upregulated SDF-1. Therefore, IL-10-expressing MSCs may have potential applicability through IL-10 paracrine effects involving SDF-1 [68].

Hepatocyte growth factor (HGF) exhibits neuroprotective effects, which may also inhibit the disruption of the blood–brain barrier after cerebral ischemia by gene administration [79]. As the stability and half-life of the translated protein and gene expression vector are limited in vivo, ex vivo therapy may exert protective effects through the secretion of factors from the cell source. We examined whether an HGF-expression system of AAV vectors could increase the typical survival of DPSCs by exerting a paracrine effect after transplantation in a rat model [35]. Following transient middle cerebral artery occlusion (tMCAO), treatment with HGF/AAV-transduced DPSCs (HGF-DPSCs) was more effective than with untransduced DPSCs, as demonstrated by the attenuation of brain damage and improvement in neurological recovery.

The HGF-DPSC treated group contained significantly lower proinflammatory cytokines, TNF-α and IL-1β levels in the brain tissue and blood than those in the vehicle and untransduced DPSC groups at 1 and 3 days after tMCAO. HGF-DPSC treatment led to a greater reduction in the infarct volume and more significant improvements in motor function, such as rotarod and grip strength, compared with untransduced DPSCs, 7 days post-tMCAO, and this was associated with the recovery of neurological scores. Improvements in neurohistopathological analysis were supported by a significantly lower number of Iba-1-positive cells in the HGF-DPSC treated group compared with those in the untransduced DPSC group. A significant increase in the expression of tight junction proteins (ZO-1 and occludin) was observed in the microvessel area of the HGF-DPSC-treated group compared with those in the vehicle and untransduced DPSC groups, suggesting that disruption of the blood–brain barrier was ameliorated in HGF-DPSC-treated rats [35]. Thus, it is possible to promote angiogenesis and protect the brain, although this has not been confirmed using conventional DPSCs after cerebral ischemia.

HGF-DPSCs, produced by an adenoviral vector, were found to enhance IL-6 production, cell proliferation, and resistance to apoptosis in synovitis, and demonstrated therapeutic effects in rheumatoid arthritis [67]. Adenoviral vector-mediated HGF-DPSCs, which are transient expression systems, express high levels of osteogenic-related genes and exert strong osteogenic differentiation capacities, indicating that these DPSCs have more benefits in the treatment of osteoporosis [80]. Furthermore, HGF-DPSCs transduced by a lentiviral vector were shown to facilitate repair in ulcerative colitis through increased TGF-β and IL-10, reduced proinflammatory cytokines, TNF-α, and IFN-γ, and decreased expression of malondialdehyde and myeloperoxidase, which indicates an oxidative stress response [65].

There is clinical interest in the applicability of genetically modified DPSCs in ex vivo cell therapy owing to their anti-inflammatory properties and ability to release cytokines into the surrounding environment, which mediate the paracrine effects and, thereby, modify the developmental fate of neighboring cells.

## 5. Clinical Studies of DPSCs for the Treatment of Stroke

### 5.1. Overview: Clinical Trials of Stem Cell Therapy for Stroke

Stem cell-based therapy is attracting attention as an alternative treatment to improve the quality of life after stroke while receiving standard treatment for stroke [59,81,82,83,84,85]. In 2005, Kondziolka et al. reported the results of a phase 2 trial with LSB neurons (human teratocarcinoma cell line origin, Layton BioScience, Inc. Sunnyvale, CA, USA) [86]. In the same year, Savitz et al. reported the results of an open-label trial of stereotactic transplantation with LGe cells (fetal porcine striatum-derived cells, Genvec, Inc. Gaithersburg, MD, USA) in five patients with basal ganglia infarcts and stable neurological deficits [87]. The number of clinical trials investigating stem cell therapy for stroke has been gradually increasing. A comprehensive search of the ClinicalTrials.gov database was performed using the search criteria “ischemic stroke” and “stem cell” on 2 December 2021. A total of 59 results were obtained. Autologous bone marrow mononuclear cells account for the largest portion of cells, followed by autologous bone marrow stem/stromal cells. Small numbers of other cell sources, including adipose-derived stem cells or neural stem cells, have also been used. Doses of cells, which differ widely, range from 1 × 10^6^ to 1 × 10^9^, according to the route of transplantation, including intravenous, intra-arterial, intrathecal or intracerebroventricular, and intracerebral routes. Most trials were preliminary in nature and did not include control groups, whereas some trials included control groups consisting of unblinded or blinded patients. Although assessment modalities also differ widely among trials, the modified Rankin Scale, National Institute of Health Stroke Scale (NIHSS), and Barthel index have been commonly applied. In some trials, a more detailed assessment of motor function was performed using the Fugl Meyer Assessment, and quality of life scores using the European Quality of Life5 Dimension (EQ-5D) [88,89]. Recently, the usefulness of MRI for evaluating the efficacy of stem cell therapy has been reported. For example, serial changes in metabolites, such as N-acetylaspartate, evaluated by magnetic resonance spectroscopy can be used to monitor therapeutic changes [90]. Furthermore, changes in microstructural axonal fibers in the corticospinal tract can be visualized using diffusion tensor imaging to monitor therapeutic changes [91]. 

### 5.2. Recent Clinical Trials of Stem Cells Other Than DPSCs against Stroke

Savitz et al. conducted a randomized, sham-controlled, phase II trial in which subjects received intracarotid aldehyde dehydrogenase (ALDH)-bright cells (13 and 19 days poststroke) isolated from the bone marrow of patients who had recently experienced an ischemic middle cerebral artery stroke [14] (ClinicalTrials.gov, number NCT01273337). Although the study provided a framework for conducting randomized, sham-controlled trials involving the intracarotid administration of autologous-sorted BMSCs in patients with a recent stroke, there were no significant differences between groups based on any of the efficacy measures (mRS at 90 days, disability assessed by Barthel index score, EQ-5D score, and rehabilitation utilization at 1 year).

TREASURE is a randomized, double-blind, placebo-controlled, multicenter phase 2/3 trial [15], that will be conducted at 31 sites in Japan. Patients with acute ischemic stroke, including motor or speech deficits defined by the National Institution of Health Stroke Scale (NIHSS) score of 8–20 at baseline will be randomized 1:1 to receive a single intravenous infusion of MultiStem^®^ or placebo within 18–36 h of the onset of stroke. The primary outcome will be the proportion of patients with an excellent outcome at Day 90 defined by functional assessment as follows: modified Rankin Scale ≤ 1, NIHSS score of ≤1, and Barthel Index score ≥ 95.

The PISCES 2 study (ClinicalTrials.gov, number NCT02117635) was a prospective, multicenter, single-arm, open-label study in adults over 40 years of age who experienced significant upper limb motor deficits 2–13 months (median: 7 months) after ischemic stroke [16]. Twenty-three patients received transplantation of 20 million allogeneic human neural stem cells, CTX0E03, into the ipsilateral putamen. No safety concerns related to the cells were reported after 1 year. One patient demonstrated improved Action Research Arm Test performance at 3 months, and three patients improved after 6–12 months. Seven patients presented improvements in mRS scores by at least 1 point. In total, 15 patients presented improvement on one or more clinical scales [16]. A phase III prospective, randomized, controlled, double-blinded study (PISCES 3) treating 130 patients, 6–12 months poststroke, is currently ongoing in the USA.

Recently, multilineage-differentiating stress-enduring (Muse) cells, which are a distinct subset of MSCs found sporadically in the connective tissue of organs, have attracted attention as a novel stem cell therapy for the treatment of stroke [92,93,94]. In mouse models of lacunar stroke, injected Muse cells were shown to migrate to the peri-infarct brain, resulting in functional recovery and long-term integration into the host neural network [95]. Further preclinical studies designed to elucidate the interactions between Muse c and host cells may reveal restoration of the structure, vasculature, and blood–brain barrier [95,96,97]. Clinical trials using intravenous injections of donor-derived Muse cells (clinically termed CL2020) are underway for the treatment of stroke (JAPIC ID: JapicCTI-184103. A randomized, double-blind, placebo-controlled clinical study of CL2020 in ischemic stroke patients).

### 5.3. Clinical Trials of DPSCs for the Treatment of Stroke

To the best of our knowledge, there are two clinical trials on the use of DPSCs against ischemic stroke, including ours. The Open study of dental pulp stem cell Therapy in Humans (TOOTH) is a first-in-human study examining the safety and feasibility of autologous adult DPSC in stroke survivors with moderate-to-severe disability [98]. It is an open-label, nonrandomized study with a pragmatic design comprising three stages. Stage 1 involves the selection of 27 participants with MCA ischemic stroke and the commencement of autologous DPSC isolation, growth, and testing in sequential cohorts. Stage 2 involves the transplantation of ascending doses of DPSC l in each cohort, followed by a 6-month observation period to identify DPSC cell-related adverse events. Stage 3 will investigate neurosurgical intervention with the maximum tolerated dose of autologous DPSCs followed by 9 weeks of intensive task-specific rehabilitation.

The study will include participants with a stable level (modified Rankin Score [mRS] of 2–4) of chronic motor, sensory, and/or language disability for at least 6 months prior to selection. Dominant hemisphere MCA stroke survivors with aphasia will be required to attain an aphasia quotient score of 33–70 on the Western Aphasia Battery (WAB-AQ). Table 2 presents an overview of the key inclusion criteria for the present study. The primary endpoint is the safety and feasibility of intracranial administration of autologous human adult DPSCs in patients with chronic stroke and determination of the maximum tolerated dose in human subjects. Secondary outcomes include determining the effects of stem cell therapy in combination with rehabilitation to design a future phase 2/3 clinical trial. The results of this study are expected to be published in February 2022.

Administration of DPSCs intravenously is less invasive than IC transplantation. Clinical studies involving the intravenous injection of DPSCs are underway in Japan. JTR-161 is a novel allogenic human cell product comprising DPSCs isolated from the extracted tooth of a healthy adult, and is under development for cell-based therapy of ischemic stroke. To evaluate the safety and efficacy of JTR-161 in patients with acute ischemic stroke (AIS) (ClinicalTrials.gov Identifier: NCT04608838), this randomized, double-blind, placebo-controlled, multicenter clinical trial conducted in Japan was named J-REPAIR. Table 3 presents an overview of the key inclusion and exclusion criteria for this study. Patients with a clinical diagnosis of anterior circulation AIS accompanied by a National Institution of Health Stroke Scale (NIHSS) score of 5–20 at baseline will be enrolled. Patients are eligible if they receive recombinant tissue-type plasminogen activator and/or mechanical thrombectomy, or if they are not receiving either treatment. This protocol may be influenced by the fact that, as recommended by STAIR X, in patients with large vessel occlusion, in addition to thrombolytic therapy with t-pa, with the recent advent of mechanical thrombectomy, cell products should be developed in combination with thrombectomy as well as neuroprotective agents, and the recommendation was made to apply treatment immediately after reopening [99].

This study included three patient cohorts. Cohorts 1 and 2 enrolled eight subjects per cohort (JTR-161, *n* = 6; placebo, *n* = 2) to evaluate for safety. Cohort 3 enrolled 60 subjects (JTR-161, *n* = 30; placebo, *n* = 30) to enable the design of further clinical trials subjects based on safety and efficacy data. In Cohort 1, 1 × 10^8^ cells and in Cohort 2, 3 × 10^8^ cells were sequentially administered to patients. Cohort 3 received the higher tolerated dose of the two cohorts (1 × 10^8^ cells or 3 × 10^8^ cells) as determined by the recommendations of the Data and Safety Monitoring Board (DSMB). The STEPS4 report also recommended dose escalation studies, and we believe that we used a protocol in accordance with this recommendation [100]. The primary endpoint was the proportion of patients who achieved an excellent outcome at day 91 in cohort 3—defined as a modified Rankin Scale ≤ 1, NIHSS ≤ 1, and Barthel Index ≥ 95. In addition, various cytokines/growth factors before and after transplantation of DPSCs were measured to investigate the mechanism of action of DPSCs. Patients were recruited from 29 stroke centers in Japan between December 2018 and October 2021. The enrollment of patients in the study has now been completed, and the study is in the follow-up period.

## 6. Perspective

DPSCs are widely available, easily accessible, and can support well-established stroke therapies. As such, they have the potential to extend the therapeutic time window and/or augment therapeutic impact. In addition, DPSCs exhibit a higher proliferation rate, higher expression of trophic factors, and stronger neuroprotective effects and neuro-supportive properties in vitro and in vivo than MSCs, which demonstrates their potential for use in stroke treatment. Clinical challenges may include complicating factors such as the effects of age, comorbidities, timing of administration, stroke subtype, and stroke severity, all of which can affect the efficacy and safety of cell therapy. Autologous transplantation of somatic-derived stem cells is difficult for middle-aged and elderly people, but for DPSCs, the use of dental pulp banks may be an option from a young age in the future. In recent years, the use of culture supernatants and exosomes, which are extracellular vesicles released from stem cells, will also be required for cell-free therapy.

In order for pulp stem cell therapy to be widely used in clinical practice for stroke treatment in the future, it will be essential to link basic science and clinical practice, and to balance two competing parameters: quality and cost. Most importantly, however, the usefulness of stem cell therapy should be judged by neurologists and neurosurgeons involved in the actual front-line stroke care.

## Figures and Tables

**Table 1:** Experimental studies using dental pulp stem cells (DPSC) for the treatment of ischemic brain injury in animal model.

Cell Type	Number of Cells	Animal Model	Delivery Timing	Delivery Route	Results	Reference
Porcine dental pulp side population, progenitor cells	1 × 10^6^	Rat MCAO (2 h)	24 h postischemia induction	Intracerebral	Recovered motor function and infarct volume Differentiation of endogenous neuronal progenitor cells and induction of vasculogenesis	Sugiyama et al. 2011 [28]
Human DPSCs	6 × 10^5^	Rat MCAO (2 h)	24 h postischemia induction	Intracerebral (striatum and cortex)	Differentiation into astrocytes Neuroprotection Functional outcomes	Leong et al. 2012 [29]
Human DPSCs	4 × 10^6^	Rat MCAO (2 h)	24 h postischemia induction	Intravenous (tail vein)	Reduced infarct volume Improved neurological outcome Differentiation into astrocytes and neuron-like cells Promotion of angiogenesis and inhibition of astrocytes	Song et al. 2017 [30]
Rat dental pulp-derived neurospheres	1 × 10^6^	Rat severe forebrain ischemia (11 min)	3 h postischemia induction	Intravenous (tail vein)	Increased survival rate Improved cognitive functional recovery Reduced neuronal death	Kumasaka et al. 2017 [34]
Human DPSCs	1 × 10^6^	Rat MCAO (90 min)	Immediately or 3 h postischemia	Intravenous (tail vein)	Reduced infarct volume Improved neurological outcome Inflammation modulation	Nito et al. 2018 [27]
Human DPSCs, HGF-transfected DPSCs	1 × 10^6^	Rat MCAO (90 min)	Immediately postischemia	Intravenous (tail vein)	Reduced infarct and edema volume Improved neurological outcome Inflammation modulation Promotion of angiogenesis	Sowa et al. 2018 [35]
Rat DPSCs, combination with BDNF	1 × 10^7^	Rat MCAO (2 h)	24 h postischemia induction	Intravenous (tail vein)	Reduced infarct and edema volume Differentiation into neuron-like cells	Zhang et al. 2018 [32]
Rat DPSCs, combination with BDNF	1 × 10^6^	Rat MCAO (2 h)	24 h postischemia induction	Intravenous (tail vein)	Reduced infarct and edema volume Improved neurological outcome Differentiation into neuronal progenitor and neuron-like cells Triggered neurogenesis	Zhang et al. 2018 [31]
Human DPSC, PDLSCs	1× 10^6^	Rat MCAO (2 h)	24 h postischemia induction	Intravenous (tail vein)	Reduced cerebral infarct size Recovery of neurological function	Wu et al. 2020 [33]
Human DPSCs	4 × 10^5^	Rat photothrombosis induces permanent focal ischemia	3 days postunilateral photothrombotic stroke induction	Intracerebral	Success in a skilled forelimb reaching test Decreased reactive astrogliosis	Yew et al. 2021 [36]
Human DPSCs Induced neural cells	1 × 10^4^ 1 × 10^5^ 1 × 10^6^	Murine MCAO	5 days postischemia induction	Intravenous	Functional recovery Differentiation into neurons and glia cells	Matsumura et al. 2021 [37]
Human DPSCs Derived Exosome		Mice MCAO (2 h)	4 h postischemia induction	Intravenous	Reduced infarct and edema volume Improved neurological outcome Inflammation modulation	Li et al. 2021 [38]

DPSCs: dental pulp stem cells; MCAO: middle cerebral artery occlusion; HGF: hepatocyte growth factor; BDNF: brain-derived neurotrophic factor; PDLSCs: periodontal ligament stem cells.

**Table 2:** Key inclusion criteria (TOOTH study).

1. MCA ischemic stroke
2. Moderate severity chronic disability—stable level (a modified Rankin Score of 2–4) of chronic motor, sensory, and/or language disability for at least 6 months prior to selection. Dominant hemisphere MCA stroke survivors with aphasia are required to attain an aphasia quotient score of 33–70 on the Western Aphasia Battery (WAB-AQ) to participate
3. Good cognitive function—the participant must achieve a Mini Mental State Examination score of 24 or more. Participants with aphasia must score above 23 on the Raven’s Colored Progressive Matrices
4. All participants must pass a Mini International Neuropsychiatric Interview; in those with aphasia, a score of less than 17 on the Stroke Aphasia Depression Questionaire-21 is required
5. Healthy teeth to grow sufficient autologous dental pulp stem cells

MCA, middle cerebral artery.

**Table 3:** Key inclusion and exclusion criteria (the J-REPAIR study).

Inclusion Criteria
1. Clinical diagnosis of anterior circulation ischemic stroke by nuclear MRI or computed tomography
2. NIHSS score ≥ 5 to ≤20 at screening
3. Onset of ischemic stroke must have occurred within 48 h prior to the start of administration of the study product
4. A modified Rankin Scale of 0 or 1, by either self-report or family report, prior to the onset of ischemic stroke
Exclusion criteria
1. Presence of intracranial hemorrhagic change diagnosed by MRI, which is judged to be clinically important by the investigator at screening
2. Alzheimer’s disease or other dementia, Parkinson’s disease, or any other neurological disorder
3. Planned revascularization treatment including carotid endarterectomy and stenting, by the end of the evaluation (Day 91)
4. After eligibility assessment at screening, the investigator will assess NIHSS again ≥4 h after the assessment at screening. Subjects meeting one or more of the following criteria will be excluded: NIHSS score < 4 or ≥21; change in NIHSS score from screening ≥ 5.

MRI, magnetic resonance imaging; NIHSS, National Institutes of Health Stroke Scale.
